# The mental health impact of multiple deprivations under protracted conflict: A multi-level study in the occupied Palestinian territory

**DOI:** 10.1371/journal.pgph.0001239

**Published:** 2022-12-07

**Authors:** Weeam Hammoudeh, Suzan Mitwalli, Rawan Kafri, Tracy Kuo Lin, Rita Giacaman, Tiziana Leone

**Affiliations:** 1 Institute of Community and Public Health, Birzeit University, Birzeit, Palestine; 2 Department of Social and Behavioral Sciences, Institute for Health & Aging, University of California, San Francisco, California, United States of America; 3 Department of International Development, London School of Economics and Political Science, London, United Kingdom; Universitá Cattolica del Sacro Cuore, ITALY

## Abstract

Building on the literatures examining the impacts of deprivation and war and conflict on mental health, in this study, we investigate the impact of different forms of deprivation on mental health within a context of prolonged conflict in the occupied Palestinian territory(oPt). We expand the operationalization go deprivation while accounting for more acute exposures to conflict and political violence and spatial variations. We use multilevel modelling of data from the Socio-Economic & Food Security Survey 2014 conducted by the Palestinian Central Bureau of Statistics, which included a sample size of 7827 households in the West Bank(WB) and Gaza Strip(GS). We conduct the analysis for the combined sample, as for the WB and GS separately. We use a General Health Questionnaire-12 (GHQ12) score as our main outcome measure of poor health. We used various measures of deprivation including subjective deprivation, material deprivation, food deprivation, and political deprivation. In addition to the different measures of deprivation, we included acute political, health, and economic shocks in our analysis along with background socio-demographic characteristics. The results indicate significant variance at the locality level. We find a significant association between poor mental health and subjective, economic, political, and food deprivation; health, economic, and political stressors; age, and being a woman. Post-secondary education and wealth have a significant inverse association with poor mental health. Subjective deprivation is the strongest predictor of GHQ12 score in the models whereby people who feel very deprived have GHQ12 scores that are almost 4-points higher than people who do not feel deprived. Economic conditions, particularly subjective measures, are significant predictors of mental health status. Our findings confirm that political and social factors are determinants of health. Feeling deprived is an important determinant of mental health. The community effect suggests that spatial characteristics are influencing mental health, and warrant further investigation.

## Introduction

While the sight of injury, death, and destruction in conflict and violence provoke immediate and intense reactions, conflicts, and especially prolonged conflicts, impact individuals and populations in important and oftentimes less visible ways in the long-term. A key area of research examines the effects of war and conflict on mental health and wellbeing. Researchers draw on a myriad of approaches to examine the effects of exposures to conflict and political violence on mental health outcomes, including distress, anxiety, post-traumatic stress disorder (PTSD). Many of these approaches are focused on the direct impacts of violence exposure, where an expanding body of research points to the wide scale impacts of conflict on the very foundations of society, posing threats to human security, health, and wellbeing [1–5]. The literature highlights that in addition to the direct consequences of conflict exposures, conflicts strain and alter social, physical, economic, political, and environmental structures that in turn have adverse effects on health [3, 6, 7].

These studies have expanded our understanding the impacts of conflict on health in important ways, including the acknowledgment of direct and indirect pathways, and shifting the focus to daily stressors that may have greater and longer effects on mental health compared with exposure to more acute forms of violence [2–4]. In this study, we build on this growing literature in a context of protracted conflict, the occupied Palestinian territory (oPt). Specifically, we investigate the impact of different forms of deprivation on mental health within a context of prolonged conflict, while also accounting for more acute exposures to conflict and political violence and spatial variations within the oPt. We draw on Sen and Naussbaum’s capabilities approaches [8–10] and Miller and Rasmussen’s [3] ‘daily stressors’ to expand our conceptualization and operationalization of deprivation, while accounting for more acute political and other shocks. Given the growing literature that has highlighted the importance of taking into account spatial variation in terms of both exposure to political violence as well as living conditions, we apply a multilevel approach to account for spatial variations at the locality level within the oPt.

In the following sections, we begin with a brief discussion of the relationship between deprivation and mental health. We then focus on deprivation and mental health in the context of conflict–drawing on debates related to the conceptualization of deprivation–before outlining our operationalization of deprivation within the context of protracted conflict in the oPt and presenting our findings.

### Deprivation and mental health

While there is consensus on the negative impacts of deprivation on mental health [11–14], the mechanisms by which deprivation impacts mental health as well as in the conceptualization of deprivation itself are heavily debated. Much of the research evidence conceptualises deprivation in economic terms, focusing on income-deprivation or other forms of material deprivation (eg: asset-deprivation) [9, 11]. Such measures of deprivation largely focus on individuals or households and do not take into account broader conditions and living standards, which may capture a fuller range of the effects of deprivation on health.

Sen [9, 10] shows the limitations of focusing on poverty or deprivation solely in terms of income-deprivation, which ignores the fact that individual characteristics (e.g., disability) and contextual characteristics (e.g., conflict) may interact to influence health. Alternatively, he proposes conceptualizing poverty as capability deprivation. Sen’s conceptualization [10] provides a useful starting point and links to a growing body of public health evidence in which relative measures of deprivation are taken into account in examining the relationship between deprivation and health [11]; here deprivation is viewed more broadly in terms of its effect on increasing vulnerabilities or reducing adaptive capacities [15, 16], which in turn can reduce opportunity and adversely affect health and wellbeing. These literatures highlight the importance of rethinking relative deprivation in terms of either social comparisons or the social distance that is created or expounded by inequalities [11, 14], which then negatively impact health through various mechanisms, including limiting access to health and social services and reducing psychosocial wellbeing [11, 12, 17]. The evidence illustrates that relative deprivation can predict poor mental health [18], depressive symptoms [12], psychological stress and poor self-rated health [19]. Similarly, previous studies noted the importance of the subjective assessment of relative deprivation, emphasizing how these subjective ratings are more strongly associated with poor physical and mental health [11], and depressive symptoms [12] than objective ratings.

### Deprivation and mental health in conflict settings

Expounding on the relationship between conflict and mental health, an increasing body of literature has pointed to the ways in which conflict works to affect what Miller and Rasmussen [3, 4] term ‘daily stressors’, that in turn affect health. These daily stressors are “the stressful social and material conditions of everyday life that are common within settings of organised violence” (4, p.33). These conditions include poverty, social marginalization, inadequate living conditions, and changes in family structure and social functioning [3], which in turn adversely affect health and wellbeing. In a review of studies that drew on their initial framework to examine the effects of daily stressors on mental health in war and conflict contexts, Miller and Rasmussen [4] note that “much of the distress observed among war affected populations may, in fact, not be due to exposure to political violence *per se*, but to its ongoing impact on multiple domains of people’s lives” (p.34). Subsequent studies have incorporated ‘daily stressors’–which include material conditions, political conditions, and lack of freedom into their frameworks or approaches, and show that political violence stemming from conflict affect people’s daily life conditions and adversely impact mental health [20].

These daily stressors, while not necessarily framed in terms of deprivation, contain considerable overlap with conceptualizations of deprivation, especially in terms of poverty and living conditions. The literature discussing the relation between deprivation and mental health is sparse in low-income settings and particularly in conflict-affected areas [21, 22]. Two studies discuss the relation between deprivation and health outcomes among Palestinians living in refugee camps in Lebanon. One study highlights that material deprivation based on objective measures can predict poor self-rated health among Palestinian refugees [22], while the other study emphasizes that relative deprivation, and not the absolute measures of socioeconomic status, is a strong predictor of both self-rated health and self-reported chronic diseases among Palestinian refugee women [21]. Likewise, evidence from a study conducted with Syrian refugees living in Jordan shows that experiencing employment deprivation can negatively impact their health [23]. Researchers in Colombia [2] also demonstrate that structural violence is linked with economic deprivation, inequalities, and insufficient job opportunities. Furthermore, they point to inequalities in exposure to violence and their impacts during conflict, can have far-reaching and intergenerational impacts on living conditions and mental health.

This growing literature provides important insights into how various forms of stressors or deprivations can be exacerbated in conflict settings and how multiple forms of deprivation can overlap in people’s lives and interact with other disadvantages, with important implications for their health and wellbeing. These studies point to a widening scope in how deprivation is conceptualized, which has been echoed in more recent trends in multidimensional poverty and deprivation measurement [24–26]. Furthermore, they point to the importance of not only including a broader range of dimensions of deprivation spanning from the political to the economic, but also using measures that are meaningful to people and the contexts in which they live [2, 4, 11, 17, 20, 27–29]. As Sen reminds us, “human lives are battered and diminished in all kinds of different ways, and the first task in this perspective, is to acknowledge that deprivations of very different kinds have to be accommodated within a general overarching framework.” And that this framework “…must not try to overlook the pluralities that are crucially involved…(9 p.19)”.

The aim of this study is to contribute to this growing literature by examining the effects of various forms of deprivation on mental health within the context of the oPt, where protracted conflict has shaped lives, health, and livelihoods [1, 5, 30, 31]. We further develop the conceptualization of deprivation and operationalize the expanded conceptualization of deprivation. In order to cover as broad and contextually relevant a range as possible, from available data, we focus on four key dimensions of deprivation: subjective deprivation, material (economic) deprivation, food deprivation (insecurity), and political deprivation. Furthermore, we outline key areas of deprivation that are likely to be of relevance to other contexts, particularly conflict settings, and contribute to the further development of multidimensional measures of deprivation. The findings from this study will provide quantitative evidence to inform a broader conceptualization of the effects of deprivation on mental health.

## Methodology

### Study context

The West Bank (WB) and Gaza Strip (GS) are part of the oPt. While both regions have been experiencing severe and protracted political violence by the Israeli occupation, this violence has intensified in the GS since 2007 [5, 31, 32]. Israel implemented a siege on the Strip that restricts the movement of Palestinian living there in and out of GS, and continues to prohibit the entrance of many goods [32]. The continuous blockade on GS has negatively affected the economy and worsened the living conditions the GS [31, 32]. The unemployment rate in the GS reached 45% compared to 17% in the WB, 76.9% of the households in GS received some kind of assistance compared to 16.7% in the WB, 54.2% of the households in GS perceived themselves as poor compared to 13.6% in the WB (PCBS, 2020). Moreover, the healthcare system in the GS continues to suffer stunting, including the insufficiency of equipment and medications as a result of the Israeli siege [33].

### Data

In our analysis, we rely on secondary data from the 2014 Socio-economic and Food Security survey (SEFSec) conducted by the Palestinian Central Bureau of Statistics (PCBS) in cooperation with the Food Security Sector (FSS). The survey sample consisted of 8,177 households in the WB and the GS. Our final analytic sample consisted of 7,723 households with complete information on all the key variables of interest. The sample is representative at the governorate and type of locality levels (i.e. urban, rural, and camp localities), which are the second and third level administrative units in the oPt [32]. The survey instrument consisted of three questionnaires: the household roster, the household questionnaire, and the individual questionnaire. The household roster includes basic demographic information for each member of the household, including age, sex, educational status, and relationship to the labor force. The household questionnaire includes detailed questions on the conditions of the household, some questions pertaining to exposure to political violence for the GS sample, questions on shocks faced by the household within six-month period prior to the survey, and questions pertaining to food insecurity. The individual questionnaire was completed by one randomly selected adult 18 years or older from each of the households and includes questions pertaining to mental health, subjective health, human insecurity, and distress.

### Ethical considerations

The data that we use in this study is secondary data that we obtained in anonymized form from the Palestinian Central Bureau of Statistics (PCBS). Due to the secondary and anonymized nature of the data, this was deemed to be exempt from ethical review by the ethical review committee at the Institute of Community and Public Health at Birzeit University.

### Analytical approach

#### Dependent variable

Our key outcome variable of interest is the General Health Questionnaire 12 (GHQ12) score, a validated instrument often used as a screening tool for identifying non-psychotic and minor psychiatric disorders [34]. There are different versions of the GHQ, where the GHQ12 is the shortened form often used in research studies. The short version of the GHQ12 has been broadly used in primary health care settings and population-based surveys as a screening tool for psychological problems [35]. It has been found to be a valid and reliable instrument among different populations [34, 36–38]. Substantial epidemiological evidence has shown that income inequality and objective measures of relative deprivation are associated with poorer health outcomes. However, surprisingly little research has examined whether subjective feelings of relative deprivation are similarly linked with poorer health outcomes. The relative deprivation hypothesis suggests that inequality affects health at the individual level through negative consequences of social comparison. We directly examined the relationship between subjective feelings of personal relative deprivation and self-reported physical and mental health in a diverse community sample (n = 328). Results demonstrated that subjective feelings of personal relative deprivation are associated with significantly poorer physical and mental health. These relationships held even when accounting for covariates that have been previously associated with both relative deprivation and health. These results further support the link between relative deprivation and health outcomes and suggest that addressing root causes of relative deprivation may lead to greater individual health [11, 36, 38, 39]. Additionally, some studies have used the GHQ12 as a measure of mental health status and deprivation [40, 41].

There are different approaches to scoring the GHQ12 instrument. We opted for the Likert scale scoring technique as we are interested in mental health as an outcome [35, 37, 42]. Using this method allows us to leverage and maintain gradations in the data without transforming the data substantially. The final GHQ12 score range is 0–36, where a higher score indicates greater mental distress or poorer mental health.

#### Independent variables

Our key independent variables in the analysis are divided into two main categories: deprivation measures and acute shocks, which we outline below. In addition to these variables, we control for age, education, and household employment in the models, given that these variables have been shown to have an impact on mental health and where education and household employment also reflect socioeconomic conditions [43–45].

### Deprivation measures

#### Subjective deprivation

Mishra and Carleton [11] argue that one key dimension of deprivation is feeling deprived or subjective deprivation. For this dimension, we rely on the question “*to what extent do you feel deprived*?*”* The response set for this question in the survey consists of a 5-point Likert scale ranging from never to very much. We recoded the responses–according to severity of subjective deprivation–into the following categories: not feeling deprived, felt a little to somewhat deprived, and felt deprived a lot or very much.

#### Material deprivation

There is considerable variation in the literature regarding the measurement of deprivation, where proxies for poverty are used at times, or relative material conditions are used [11, 12, 18, 28]. Here we use both ‘subjective’ and ‘objective’ measures of material conditions. For the subjective measure, we rely on one question asking respondents to rate their economic status, with a possible range of responses from very poor to rich. We also created a composite ‘wealth’ score using principle component analysis taking into account household material conditions and amenities [46, 47]. We then classified respondents into wealth quartiles, in order to take into account where they stand in relation to others.

#### Food deprivation

Linked to material deprivation, we operationalize food deprivation using two measures of food insecurity, the Household Food Insecurity Access Scale (HFIAS) and household food consumption score (FCS), which have both been used and validated in international contexts [32, 35, 48–50]. The HFIAS is used to assess food insecurity at the household level, which is one type of deprivation people can be exposed to. The FCS score gives us a sense of food diversity and consumption at the household level, and is also indicative of material conditions.

#### Political deprivation: Human insecurity

In the context of the oPt, while deprivation is linked to material conditions, the local understanding of deprivation is also linked with the political context. In this context, and other conflict settings potentially, the conceptualization of deprivation should take into account political conditions and the lack of freedoms [8, 10, 20].

In this study, we use human insecurity [5] as a measure of political deprivation. The human insecurity measure that we use was developed over the years, which began with qualitative work followed by survey work. The instrument has also been assessed in relation to other measures of wellbeing, including quality of life [31]. Here we operationalize political deprivation through the human insecurity scale score. An increase in the score is indicative of higher insecurity stemming from the political context. The measure was developed and validated locally [5], as part of a broader effort to develop and validate quality of life measures of relevance to the Palestinian context [30, 51], and has been used in the oPt as well as in other contexts [29]. In the Palestinian context, it has been shown to be sensitive to changes stemming from intensifications in violence [31].

### Shocks

In addition to the deprivation measures, we also account for more acute shocks in our analysis. These acute shocks represent shocks experienced by households in the six months preceding the survey, and include economic, political, and health shocks as outlined below.

#### Economic shocks

This measure is a summed count variable that includes six items: loss in assets including land and projects; inability to repay loans; loss of part or all of salary/income; delay of payment of salary; loss of some or all of assistance; and inability to pay treatment costs. Respondents were asked about whether their household experienced these shocks in the six months preceding the survey. For each of these items, a positive response was given as score of 1, and then all positive scores were summed for a total score.

#### Political shocks

For this measure, we created different measures for the WB and the GS. Given that the survey was conducted after the 2014 attack on the GS [32], specific questions about exposures to political violence were asked in the GS in an additional section of the questionnaire. In the section on shocks in the SEFSec questionnaire, three questions pertinent to the political context were asked to respondents in both the WB and the GS. The measure for the WB consisted of: loss of assets or projects due to Israeli measures; restrictions imposed on access to land; lack of permits. For the GS, in addition to these items, we counted whether any member of the household was killed in the last war; whether the household faced destruction or damage to their home; and whether at least one member of the family was injured in the last war. For the WB, the scores range from 0 to 3, and in the GS from 0 to 6. These sets of political shocks serve as a proxy for political violence exposure [33], albeit with limitations since we do not have readily available data for a broader range of potential political violence exposures.

#### Health shock

Various studies have found that health shocks within the family or household can have adverse impacts on economic conditions and may push households towards reducing spending on other essential needs, like education and food [52, 53]. Furthermore, the stress of the financial strains of health shock and the increase in worry and care-taking responsibilities can have adverse impacts on mental health and wellbeing more broadly [54, 55]. The health shock is based on one question in the SEFSec survey asking whether the a member of the household has a serious illness that inhibits performance of routine activities. If the respondent responded positively, they were given a score of one, and zero otherwise.

#### Analysis

We began the analysis with basic descriptive statistics and bivariate analysis. The GHQ12 score is the main outcome variable of interest, and since the score is a continuous variable, we use t-tests and ANOVA in the bivariate analysis. Given the differences between the two regions outlined above, we ran the analysis for both the WB and GS together, and then separately. In the multivariate analysis, we use a two-level mixed random intercept model for our analyses, with a locality variable as a proxy for neighborhood. Initially, we estimated a single level continuous regression model, then given the multilevel nature of the determinants of health outcomes and the multilevel structure of the survey we added random intercepts at the locality level. Locality is taken as a proxy for community effects as demonstrated in previous literature [46]. We first ran the aggregate model and then separate models for the WB and GS. The likelihood-ratio test confirmed the appropriateness of using the multilevel model. The random effects are not only suitable for the structure of the nested data (e.g. individuals within localities) but also key in understanding the impact of determinants on mental health at community level. Within a setting where blockades and checkpoints deny free access, it is particularly relevant to highlight the diverse extent of the issues.

The final model was as follows:

GHQscoreij=β0+β1Stressorsij+β2SESij+β3DEPij+δ0j+ℇij

Where δ0j and ℇij are the errors terms for level 1 (i) and level 2 (j), respectively. *Shocks* is the vector of shocks as described in the previous section and SES is the vector of all the other variables. SES are the socio-economic variables and DEP are the deprivation variables as described previously *i* is the individual level and j is the locality level.

Data were weighted using relative weights as reported in the survey to control for the survey design and random intercept models run in Stata 15.

### Limitations

This study utilizes data and empirical strategies, which permit the examination and expansion of deprivation as a concept and a quantitative measure, but there are several limitations to this study. Due to data availability at the time of analysis, this study only includes a cross-sectional survey dataset with one time-period, which prohibits the evaluation of causality. Our models highlight the association between our variables of interest but do not provide causal inference. There is likely an endogenous relationship between subjective deprivation and poor mental health, where people with poorer mental health are more likely to indicate subjective deprivation and vice versa. Nevertheless, demonstrating the association between our variables of interest is the first step to understanding how these variables may affect each other. The dataset is based on self-reported outcomes; however, the questions and choice responses are derived from validated instruments that have been adopted globally. Lastly, our conceptualization and operationalization of deprivation as a concept should be viewed as only a starting point to developing comprehensive measures and capture the intricate relationship between deprivation and mental health, particularly in conflict and war settings.

## Results

In the remainder of this article, we begin by presenting the results from the combined model followed by the results from the separate models. A description of the sample is reported in Table 1. We ran the overall model including both the WB and GS and fixed effects for region and found that there was significant variation between the models where each of the separate models obtained better model fit as measured by AIC/BIC. Analyzing the WB and the GS models separately thus allow us to examine closely how these factors operate within the WB and GS differently. Furthermore, the spatial component was more evident in the WB and explained a greater proportion of the variance. While the WB and GS are part of the oPt and constitute a political entity, circumstances in these two regions differ substantially between the two. Since the siege conditions in the GS were intensified in 2007, circumstances have continued to worsen substantially. This has been further exacerbated after three wars on the GS since 2008 [31, 32, 56]. These differences are important to take into account, especially given our focus on the relationship between deprivation and mental health. The relative experience of deprivation and its overall impact is likely to vary considerably between the two regions. The GS is also a smaller geographic entity with less variation between the different regions compared with the WB, which is larger and more heterogeneous. Our model results indicate that spatial variation is greater in the WB.

**Table 1:** Sample characteristics.

	COMBINED		WB		GS		RANGE	
VARIABLE	Mean	Std. dev.	Mean	Std. dev.	Mean	Std. dev.	Min	Max
GHQSCORE	9.06	5.01	7.95	4.53	10.94	5.22	0	34
Age	38.49	15.48	39.45	15.95	36.89	14.50	18	98
Household size (discrete)	5.52	2.59	5.14	2.38	6.15	2.80	1	24
Food Insecurity	2.13	2.74	1.18	2.16	3.73	2.86	0	9
Household food consumption	73.98	16.91	76.50	15.60	69.74	18.15	0	112
Human Insecurity	20.05	7.05	18.42	6.56	22.78	7.01	0	36
Shocks								
Economic shocks	0.92	1.10	0.52	0.78	1.59	1.23	0	6
Political shocks	0.20	0.45	0.15	0.37	0.29	0.56	0	3
Political shocks_Gaza					0.95	0.92	0	5
Health shock	0.033	0.18	0.031	0.17	0.038	0.19	0	1
	%		%		%			
Women	50.93%		51.02%		50.76%			
Below secondary education	60.95%		66.36%		51.84%			
Secondary education	22.89%		21.27%		25.63%			
Postsecondary education	21.68%		19.06%		26.08%			
At least one household member employed	74.52%		82.90%		60.42%			
**SUBJECTIVE DEPRIVATION**								
Not deprived	61.91%		73.45%		42.50%			
Little to moderately deprived	20.96%		17.43%		26.91%			
Very deprived	17.13%		9.13%		30.59%			
**SUBJECTIVE SES**								
Rich (good)	3.03%		3.42%		2.40%			
Middle class	70.23%		74.06%		58.75%			
Poor	21.44%		17.23%		28.51%			
Very poor	7.17%		5.28%		10.35%			
	n = 7723		n = 4843		n = 2880			

### Multivariate models

We estimated the single-level model followed by the random intercept model with the random intercept at locality level. The log likelihood test ratio showed that in all four models the model fit improved where the LR test was significant at the p<0.001 level across all models when the random intercept was included. The multilevel models (Table 2) show a significant variance at locality level across all models. The greatest variance is at locality level across all models. More specifically the level of variance explained by locality is 7.5% in the combined model, 11.4% in the WB model, 3.1% in the GS model and 2.1% in the GS political model. This result is not surprising given the relative homogeneity of the GS compared with WB which is characterized by greater geographic spread and a regime of fragmentation through checkpoints, illegal settlement blocks, and variable access to services.

**Table 2:** Models.

	Model 1-Combined Model			Model 2-WB model			Model 3-GS model			Model 4-GS political model		
VARIABLES	(1)	(2)	(3)	(1)	(2)	(3)	(1)	(2)	(3)	(1)	(2)	(3)
	GHQscore	lns1_1_1	lnsig_e	GHQscore	lns1_1_1	lnsig_e	GHQscore	lns1_1_1	lnsig_e	GHQscore	lns1_1_1	lnsig_e
Age	0.0892***			0.0434**			0.176***			0.174***		
	(0.0151)			(0.0171)			(0.0295)			-0.0295		
age2	-0.000477***			-1.95e-05			-0.00139***			-0.00138***		
	(0.000166)			(0.000186)			(0.000333)			-0.000332		
Women	0.251***			0.485***			-0.191			-0.178		
	(0.0912)			(0.105)			(0.167)			-0.167		
Below Secondary	0.0911			0.162			0.0787			0.07		
	(0.107)			(0.125)			(0.193)			-0.193		
Post-Secondary	-0.576***			-0.558***			-0.780***			-0.754***		
	(0.126)			(0.150)			(0.219)			-0.219		
Econ Shock	0.227***			0.380***			0.144*			0.141*		
	(0.0529)			(0.0792)			(0.0751)			-0.0743		
Political Stress	0.522***			0.472***			0.497***			0.449***		
	(0.108)			(0.153)			(0.157)			-0.0964		
Health Shock	1.362***			1.085***			1.459***			1.379***		
	(0.262)			(0.323)			(0.443)			-0.443		
Subjective wealth-middle is reference												
Rich	-0.660**			-1.213***			0.676			0.694		
	(0.264)			(0.282)			(0.569)			-0.568		
Poor	0.650***			0.819***			0.555**			0.550**		
	(0.145)			(0.200)			(0.218)			-0.217		
Very Poor	2.288***			3.031***			2.098***			2.024***		
	(0.239)			(0.393)			(0.330)			-0.33		
Income Quartiles												
Poorest quartile	0.854***			0.805***			0.758**			0.775**		
	(0.168)			(0.211)			(0.325)			-0.324		
Second Quartile	0.543***			0.503***			0.306			0.321		
	(0.146)			(0.161)			(0.319)			-0.318		
Third Quartile	0.516***			0.419***			0.488			0.48		
	(0.133)			(0.138)			(0.330)			-0.329		
Human Insecurity	0.0432***			0.0402***			0.0532***			0.0549***		
	(0.00752)			(0.00917)			(0.0129)			-0.0128		
FoodInsecurity	0.187***			0.287***			0.103***			0.0899**		
	(0.0241)			(0.0314)			(0.0378)			-0.0378		
Food Consumption	-0.0126***			-0.0154***			-0.00896*			-0.00968*		
	(0.00311)			(0.00387)			(0.00514)			-0.00513		
At least one household member employed	-0.377***			-0.332**			-0.309*			-0.320*		
	(0.120)			(0.169)			(0.177)			-0.177		
Household_Size	0.0424**			0.0320			0.0639*			0.0583*		
	(0.0206)			(0.0263)			(0.0332)			-0.0332		
Subjective Deprivation-not deprived is ref												
Somewhat Deprived	1.692***			1.271***			1.708***			1.687***		
	(0.134)			(0.152)			(0.211)			-0.21		
Very Deprived	4.225***			3.939***			3.676***			3.627***		
	(0.209)			(0.212)			(0.216)			-0.216		
GS	0.648**											
	(0.292)											
GS dep interaction	-0.381***											
	(0.132)											
Constant	3.869***	0.120	1.376***	4.913***	0.253***	1.280***	2.767***	-0.238	1.487***	2.607***	-0.249	1.485***
	(0.464)	(0.0823)	(0.00814)	(0.549)	(0.0821)	(0.0103)	(0.865)	(0.213)	(0.0132)	-0.864	-0.211	-0.0132
Observations	7,723	7,723	7,723	4,843	4,843	4,843	2,880	2,880	2,880	2880	2880	2880
Number of groups	199	199	199	169	169	169	30	30	30	30	30	30

Standard errors in parentheses.

*** p<0.01,

** p<0.05,

* p<0.1.

Overall, in all the multivariate models and analyses the variable that has the most impact on GHQ12 score (poor mental health) is subjective deprivation. People who reported some deprivation have scores that are about 1.69 points higher compared with those who have not reported any deprivation; among people who are very deprived, the score increases by 4.23 points. This pattern is statistically (p<0.001) and substantively significant in terms of magnitude. In terms of the other forms of deprivation, material deprivation is the second most important predictor in the overall model and in the WB model (this would include subjective economic status, wealth quartile, and economic shocks), then the political, and finally food deprivation (insecurity and consumption). In terms of the political, experiences of political shocks and greater human insecurity are associated with worse mental health (GHQ12 increases by 0.52 per political shock and 0.043 per point on human insecurity scale, both significant at p<0.001). Women have higher scores than men, older people have higher scores than younger people, and people with post-secondary education have lower scores than people with secondary education. People who reported the health stressor (one item consisting of having an illness that limits function) also have higher scores on the GHQ12 across models.

### Combined model

The first set of analysis included combined the samples for WB and GS (GS) as shown in Table 2. Subjective deprivation has the largest effect on GHQ12 score. The results indicate that people who have some deprivation have scores that are about 1.69 points higher compared with those who don’t have any deprivation (p<0.01). The score among people who are very deprived is 4.23 points higher compared with not deprived (p<0.01). Material deprivation consisting of subjective economic status classified as rich, middle, poor or very poor; wealth quartile variable (poorest quartile, second quartile, third quartile and the richest quartile); and economic shocks are the second set of determinants of poor mental health. People who perceived themselves as very poor have about 2.29 points higher GHQ12 score compared to people who identified as middle class (p<0.001). People who perceived themselves as poor have an increase in the score by 0.65 points (p<0.001) also in comparison with middle wealth people, and those who reported themselves as rich have 0.66 (p<0.01) lower score of GHQ12. The scores of GHQ12 for poorest quartile, second quartile, and third quartile increase by 0.76, 0.48 and 0.49 (p<0.01) respectively compared to the richest quartile. People suffering from economic shocks have an increase in GHQ12 score by 0.23 points (p<0.001) per shock. Food insecurity has a significant positive association with GHQ12 score (β = 0.187, p<0.001), while more food consumption is associated negatively with GHQ12 score (β = -013, p<0.001). In other words, food insecurity is associated with poorer mental health, while greater food consumption is associated with better mental health.

People who have experienced political shocks have worse mental health, where each experience is associated with about an 0.52 point increase in GHQ12-score (β = 0.52, p<0.001). Similarly, greater human insecurity slightly increases GHQ12-score by 0.043 points (p<0.001). The health stressor (one item which is having an illness that limits function), is another predicting factor for mental health with those who reported having the health stressor have 1.36 points higher GHQ12 score compared to those who did not (p<0.001). Age is positively associated GHQ12 score, where each year of age is associated with about a 0.1 increase in score (β = 0.089, p<0.001), i.e. older age is associated with poorer mental health. Women have higher scores than men (β = 0.25, p<0.001), and people who have post-secondary education have significantly lower scores than people with secondary education (β = -0.57, p<0.001). People who reported having one household member or more employed have lower GHQ12 scores (β = -0.38 p<0.001) compared to those who do not have employed members.

### Separate models of the WB and GS

As in the combined model, subjective deprivation is the strongest predictor of GHQ12 score in the models for the WB and GS, with similar coefficient sizes. People who feel very deprived have GHQ12 scores that are almost 4 points higher than people who do not feel deprived (β = 3.94 in the WB (model-2) and 3.63 in the GS (model-4), p<0.001). Upon closer examination of the various dimensions of deprivation, we find that, in general, experiences of deprivation have an adverse effect on mental health, with some variation between the WB and GS in terms of the size of effect on GHQ12 score.

#### Material forms of deprivation: Economic dimensions and food insecurity

Economic conditions, measured through different variables, are significant predictors of mental health status. In both settings, we find that even after controlling for employment and wealth quartile, the subjective measures of economic status have a stronger effect on GHQ12 score, with some variation in magnitude between the WB and GS. People who consider themselves to be very poor have scores that are 3.03 and 2.02 higher than people who consider themselves in the middle wealth status in the WB and GS, respectively (p<0.001). People who classify themselves as poor have scores 0.82 and 0.55 points higher compared with those who classify themselves as in the middle wealth status in the WB and GS, respectively (p<0.001).

In the WB, people who consider themselves rich have scores 1.21 points lower compared with those who classify themselves as middle class (p<0.001). This association is not statistically significant in the GS. In terms of the objective relative economic standing (as measured by wealth quartiles), in the GS those who are classified in the poorest quartile have scores 0.76 points higher than the richest quartile (p<0.001). The differences between the other quartiles in the GS are not statistically significantly different from the richest quartile. In the WB, all other quartiles have statistically significantly higher scores compared with the richest quartile (β = 0.81 for poorest, β = 0.50 in second quartile, and β = 0.42 in the third quartile, p<0.001). People whose household experienced economic shocks had higher scores on the GHQ12 scale. In the WB, for each reported economic shock, the GHQ12 score increases by 0.38 points (p<0.001), and for GS, the GHQ12 score increases by 0.14 points(p<0.05) per shock.

Food insecurity was positively associated with poor mental health in both the WB and GS. For each point increase in the HFIAS scale, the GHQ12 score increases by 0.29 points in the WB and 0.10 points in the GS (p<0.001). In the WB, food consumption had a protective effect on mental health, whereby each point increase on the FCS scale resulted in a 0.015-point decrease (p<0.001) in the GHQ12 score, and 0.009 (p<0.05) decrease in the GS.

#### Political deprivation and shocks

In both the WB and GS, political exposures are important predictors of mental health. Human insecurity, which is our operationalization of political deprivation, was positively associated with poor mental health. Each point increase on the human insecurity scale (out of a maximum score of 100) results in a 0.040-point increase in GHQ12 score in the WB (p<0.001) and 0.055 in the GS (p<0.001). Furthermore, people in households exposed to more acute political shocks have significantly higher GHQ12 scores. In the WB, for each reported political shock (out of 3), GHQ12 score increases by 0.47 points (p<0.001). In the GS, for each reported political shock (out of 6), GHQ12 score increases by 0.45 points (p<0.001). Therefore, someone exposed to the maximum number of measured political shocks in the WB would have a GHQ12 score about 1.5 points higher than someone not exposed to political shocks. In the GS, someone exposed to all political shocks accounted for, would have a score about 2.8 points higher than someone not exposed to any political shocks measured in the survey.

### Health stressor and sociodemographic determinants

People who reported that they have an illness that limits function have GHQ12 scores that are 1.09 and 1.38 points higher than those who did not report an illness that limits function in the WB and GS respectively (p<0.001). In terms of other sociodemographic characteristics, age is positively associated with poor mental health in both settings, although with a stronger effect in the GS (β = 0.043 in the WB and β = 0.17 in the GS, p<0.001). In both settings, post-secondary education was a protective factor against poorer mental health (β = -0.56 in WB, and β = -0.75 in the GS, p<0.001). In the WB, women had scores 0.479 points higher than men (p<0.001). Household size was positively associated with GHQ12-scores in the GS (β = 0.058 in the GS, p<0.05).

The key differences in the WB and GS appear to be in the magnitude of the effect of economic predictors, where they have a greater impact on GHQ12 score in the WB. Furthermore, in the GS, extreme poverty appears to be what counts in negatively affecting mental health. Food insecurity is significant in both settings, but greater in magnitude in the WB. Political determinants are statistically significant in both settings with similar coefficients; however, the range of scores are wider and overall scores higher in the GS. In all models we controlled for household size being both a measure of network and of burden on resources as well as a proxy for number of children often identified as a source of support. The variable was only mildly significant in the GS model.

## Discussion

This study contributes to the literature on deprivation and health on several levels. First, we expand the operationalization of deprivation beyond the economic, notably we characterize and analyze a political dimension. We conceptualize deprivation in broader terms, including subjective measures of material and economic conditions, including food security, while also taking into account absolute measures of material and economic conditions, including food consumption and wealth. Second, our study contributes to the burgeoning literature on deprivation and vulnerabilities in conflict settings by taking into account measures that reflect exposures to violence and accounting for spatial variation across localities through multilevel analysis. From an analytical point of view, this is, to our knowledge, the first study in a low-middle-income setting that uses multilevel modelling to analyze multiple dimensions of deprivation, including food insecurity. We include measures of acute political violence or shocks, like injury or home demolition, alongside more chronic measures, like human insecurity. Last but not least, this study uses the oPt as a case study and compare within and across different levels of geographical exposure to conflict and levels of deprivation.

The first key finding from our analyses is the role of the political dimensions. In our conceptualization, we operationalize political deprivation as human insecurity utilizing a locally developed measure [5] inspired by the framework for human insecurity put forth by Jennifer Leaning and colleagues. In addition to the measure of human insecurity, which can reflect a composite measure of political deprivation and has been shown to be more sensitive to changes at the population level [31], we included more acute measures of exposures to political violence for the WB and GS. Given that the survey was conducted about four months after the 2014 attack on the GS, additional exposure questions were asked in the GS, which we included in separate analysis for the GS. Our inclusion of human insecurity and acute shocks allow us to account for multiple vulnerabilities that have been shown to have compounded effects on mental health in other conflict settings [17]. Although insecurities and vulnerabilities are generally heightened in conflict and war settings, the findings from this study reaffirm Trani and Bakshi’s [17] findings, which show that considerable variability within conflict contexts exists, and pre-existing vulnerabilities, like poverty, can be extenuated by additional exposures to direct acute violence. Although we are limited by the available data, we include multiple measures of political violence and shocks. It is worth noting that this study is part of a larger multi-methods study that builds on this analysis with qualitative work in order to develop a contextually relevant conceptualization of deprivation based on local understandings [57], where our data points to a multidimensional definition that includes political, economic, and social dimensions of deprivation [58].

As others [11, 12] have shown, subjective deprivation is an important predictor of mental health status, independent of other measures of deprivation. In our analyses, subjective deprivation had the largest coefficient of any covariate included in the respective models. This finding underpins Mishra and Carleton’s argument that feeling deprived is a necessary dimension in the pathway by which deprivation affects health. Among the economic measures we include in our model, we find that both subjective and ‘objective’ measures of economic status affect mental health. Consistent with Beshai et al. [12] and Mishra and Carleton [11], subjective measures accounting for how people feel about their economic conditions and standing appear to have a greater effect than wealth quartiles, particularly amongst those who consider themselves among the poorest.

The independent effects of subjective measures of economic standing adds to the debates on the use of relative deprivation indicators rather than depending on measures of absolute income. Due to the questionable accuracy of incomes measures in low- and middle- income countries [22], we relied on household wealth alongside other economic indicators like employment. In line with the debates in the literature on relative deprivation, we used quartiles rather than wealth score in the models to account for the household’s position in terms of economic conditions, rather than absolute wealth. The findings pointing to the independent effect of subjective standing indicate that it may be important to take into account people’s assessments of their economic position, similar to the argument put forth by Mishra and Carleton (2015) in relation to including measures of subjective deprivation. With relative measures like income categories, cutoffs are often decided by researchers and may not be consistent with the points of reference used by people to evaluate their own standing. Thereby, the addition of subjective measures of economic conditions may provide additional insights, which may be especially relevant for mental and self-rated health indicators.

In addition to the more common measures of material deprivation, we included measures of food insecurity into our model. Food insecurity is connected to economic conditions and increasingly of interest in multidimensional measures of poverty and deprivation [25, 26], and has been shown to have an impact on self-rated health and mental health in other studies [59–61]. We included a measure of household food insecurity based on the HFIAS scale developed by the FAO [62]. This scale reflects experiences of anxiety around the adequacy of food in the household and at the higher end of the scale includes measures to reduce either the quantity or quality of food for some or all household members. We also included a composite measure of household food consumption, which is a score that takes into account the types of food consumed as well as their frequency on the household level, using a weighted scoring system. These measures add an important dimension when thinking about deprivation, especially given that food is a necessity and inextricably linked with economic conditions. Our findings show that reported food insecurity experience has a greater overall effect on GHQ12 score compared with household food consumption score. Food consumption score was only statistically significant in the WB and not in the GS, which may be related to less variation within the GS and generally worse conditions overall. Studies [63] have found food insecurity to be significantly associated with subjective well-being in multi-country samples, with stronger associations in more developed countries compared with less-developed [59]. Another study found persistent food insecurity to be significantly associated with worse mental health among women living with or at risk of HIV in the United States [61].

Our analyses show that there is a considerable amount of variance across space, particularly within the WB. There is a growing literature on the use of multilevel methods or area measures of deprivation [44, 64] in examining the effects of deprivation on health. In our analysis, we account for locality as a proxy for neighborhood, without including locality-level indices. The significance of the multi-level model, particularly in the WB, as well as the growing literature on the role of area level indices of deprivation in assessing the effects of deprivation on health and other outcomes, calls for new ways of examining deprivation within the oPt and other LMICs taking into account variations within settings. Incorporating such analysis may require the pooling of data from various sources in order to create area level indices, which can then be added to analyses, and potentially contributing an important component. Furthermore, the analysis in this paper points to the importance of expanding working definitions of deprivation. While theoretical arguments have been put forth calling for an expanded conceptualization and operationalization of deprivation, the literature to date largely focuses on material conditions [28]. Further work is needed on this front, and can possibly be combined with recent efforts at expanding definitions of poverty to including multidimensional poverty measures that go beyond standard economic conditions.
